# Supplementary material for: Stochastic Entanglement of Deterministic Origami Tentacles For Robust Robotic Grasping
Source: Adv Sci (Weinh). 2026 Aug 10:e76810. Online ahead of print. doi: 10.1002/advs.76810 (PMC13454981; doi:10.1002/advs.76810)
Supplement: Supplementary file 1 [file ADVS-9999-e76810-s008.pdf]

# Supplementary Materials For

# Stochastic Entanglement of Deterministic Origami Tentacles For Robust Robotic Grasping

*Alec Boron*[+] *Bokun Zheng*[+] *Ziyang Zhou*[+] *Noel Naughton* *Suyi Li*[*]

Department of Mechanical Engineering, Virginia Tech, VA, USA

[*]corresponding author, email address: suyili@vt.edu

[+]A.B., B.Z., and Z.Z. contributed equally to this work.

## Supplementary Materials

**This PDF file includes:**

Supplementary Text
Figures S1 to S7
Captions for Movies S1 to S11

**Other Supplementary Materials for this manuscript:**

Movies S1 to S11

## Section 1. Tendon Pulling Test of a Single Tentacle

We characterize single-tentacle actuation by measuring tendon force versus actuation level (tentacle % fold). Initial tests showed substantial trial-to-trial scatter even for nominally identical designs, driven in part by residual, nonuniform tension introduced during cutting and handling, which biases which joints initiate folding. To improve repeatability, we cure each tentacle immediately after fabrication by pre-folding it along the crease lines to its maximum deformation and holding it in a mold for 8 h. This conditioning relaxes asymmetric crease tension, stabilizes the effective hinge response across repeating units, and promotes more uniform folding during subsequent actuation.

For the tentacle sample with folding angles $(\alpha, \beta) = (90°, 90°)$ (T90-90), curing substantially improves repeatability: the uncured tentacle shows a broad force–fold response band, whereas the cured tentacle exhibits a much tighter band, indicating a more stable and predictable mechanical response (Fig. S1a). This reduction in scatter is consistent with the observed deformation modes, where the cured tentacle folds more uniformly along the backbone while the uncured tentacle concentrates folding in localized segments due to residual crease bias. Curing also clarifies the structure of the response curve, yielding a more distinct low-to-moderate piecewise-linear regime followed by a sharper transition at higher fold levels, where geometric instabilities and local buckling become prominent.

With curing reducing processing-induced variability, the remaining differences in force–fold response can be attributed primarily to geometry (Fig. S1b). Under the same conditioning, for the T90-90 sample, it behaves as a spring-like actuator with an approximately linear, shrinkage-dominated response over much of the stroke, consistent with a more symmetric redistribution of folding along the ribbon. In contrast, T90-75 follows a curvature-dominated pathway, producing a distinct force–fold profile and a visibly different shape evolution. At high fold levels, the two designs diverge in required actuation force by several newtons ($\Delta F \approx 2.7$ N), indicating that the cut pattern can qualitatively switch the dominant deformation mode rather than only tuning effective stiffness.

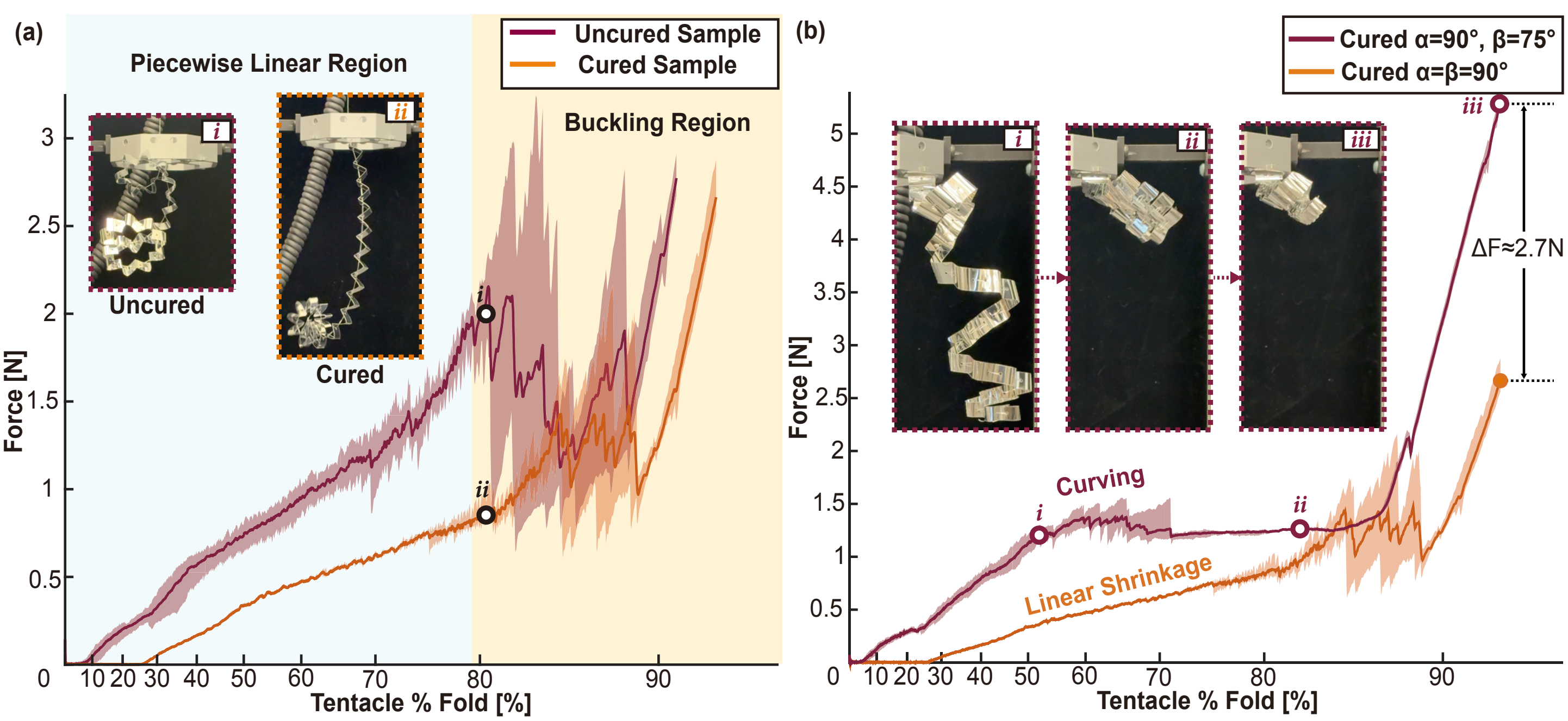


Figure S1: **Single tentacle entanglement test.** (a) T90-90 force–fold curves before/after curing, showing reduced variability after conditioning. (b) Force–fold curves for cured T90-90 and T90-75, illustrating shrinkage-dominated versus curvature/twist-biased behavior.

## Section 2. Numerical solver for origami folding angles

To efficiently compute the folding configuration, we formulate a numerical solver based on the mechanics of origami folding. Specifically, finding a scalar $k$ value so that:

$$\phi_i = k\,\frac{d_i}{w_i}, \tag{1}$$

subject to the tendon shortening length constraint

$$\sum_{i}^{N} 2d_i \cos(\phi_i) = (1-\gamma)L, \tag{2}$$

with the admissibility condition

$$\phi_i \in \left[0, \frac{\pi}{2}\right]. \tag{3}$$

where $\gamma$ is the percentage of shortening of the tendon length

Under this formulation, the original multi-variable configuration problem reduces to finding the scalar $k$ that satisfies the global length constraint. Because the left-hand side is monotonic in $k$ within, the solution can be efficiently obtained using a one-dimensional bisection root-finding procedure. Once $k$ is determined, the full set of folding angles $\{\phi_i\}$ follows directly from the linear relation above.

We additionally account for the edge case in which one or more folding angles saturate at 90° under large tendon displacements. In this regime, the single scalar gain $k$ is no longer sufficient to describe the full set of folding angles. Physically, $\phi_i = 90°$ indicates that the corresponding joint is fully folded, and the associated segment pair effectively disappears from the active kinematic chain. Based on this observation, we extend the solver to an iterative procedure. At each iteration, the algorithm detects angles that exceed the admissible range and temporarily excludes the corresponding joints from the unknown set. The scalar solve for $k$ is then performed on the reduced system. This process repeats until a feasible solution is obtained in which all remaining angles satisfy $\phi_i \in (0, \pi/2)$. The excluded joints are subsequently reinstated and assigned $\phi_i = 90°$. Algorithm 1 summarizes the pseudo-code of the full numerical solver.

We further validate the proposed solver using a physical tentacle sample with folding angles $(\alpha, \beta) = (90°, 90°)$ (T90-90), which undergoes pure 2D bending. The tentacle is captured in a static configuration on a smooth surface, with all folding joints manually relaxed to mitigate any localized stress arising from tendon loading and surface friction. The results (Fig. S2) show agreement between the predicted configuration and the measured static behavior of the physical tentacle. For larger tendon displacements (40% 50%), the proposed model continues to handle the behavior of fully folded joints as edge cases. Some discrepancy arises due to the absence of the self-contact feature, which leads to minor geometric penetration. However, our analysis primarily focuses on tendon displacements below 30%, where most tangling interactions are determined at this early stage and the model assumptions remain valid.

**Algorithm 1** Mechanics-based solver with angle saturation

**Require:** $\{d_i, w_i\}_{i=1}^N$, $\gamma$, $L$
**Ensure:** Folding angles $\{\phi_i\}_{i=1}^N$

$\mathcal{A} \leftarrow \{1, \ldots, N\}$ ▷ active joints
$\mathcal{S} \leftarrow \emptyset$ ▷ saturated joints

**while** true **do**
  Solve for $k$ such that
  $$\sum_{i \in \mathcal{A}} 2d_i \cos\left(k \frac{d_i}{w_i}\right) = (1 - \gamma)L$$
  Compute $\phi_i \leftarrow k \frac{d_i}{w_i}$ for all $i \in \mathcal{A}$
  $\mathcal{C} \leftarrow \{i \in \mathcal{A} \mid \phi_i \geq \pi/2\}$
  **if** $\mathcal{C} = \emptyset$ **then**
    **break**
  **end if**
  $\mathcal{A} \leftarrow \mathcal{A} \setminus \mathcal{C}$
  $\mathcal{S} \leftarrow \mathcal{S} \cup \mathcal{C}$
**end while**

**for all** $i \in \mathcal{S}$ **do**
  $\phi_i \leftarrow \pi/2$
**end for**
**return** $\{\phi_i\}_{i=1}^N$

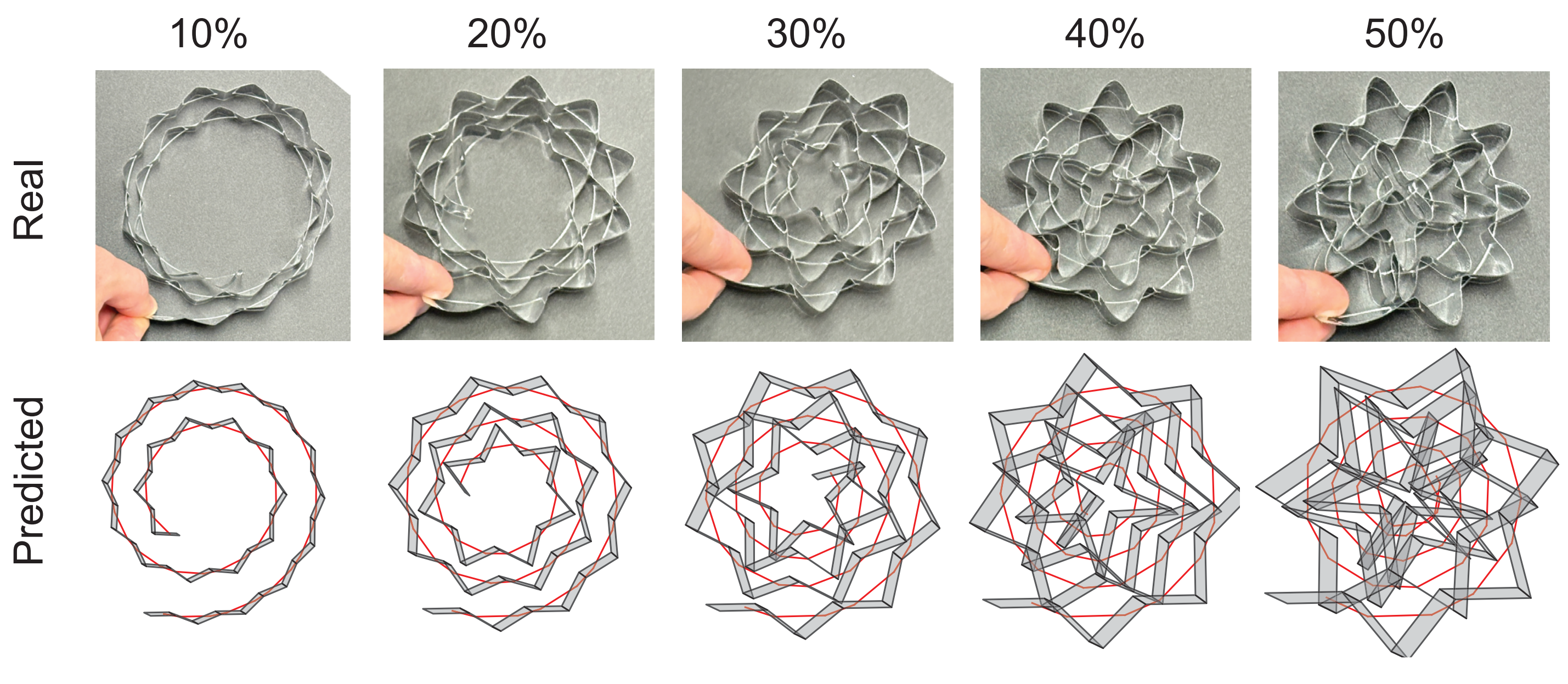


Figure S2: **Comparison between the physical tentacle and the numerical solver prediction at tendon displacements of 10%, 20%, 30%, 40%, and 50%.**

### Section 3. Evaluation metrics of simulated entanglement

With the average link numbers, the overall emergence of entangled grasping can be topologically described. Here we discuss link numbers, as well as the interactive force received by the target object, are affected by the design parameters. And how they potentially contribute to the overall performance score to better highlight high-efficient tangling. Each quantity is plotted with the same data from the parameter sweep with no gravity (Fig. 5a).

The self-linking map (Fig. S3a) shows a strong correlation with the folding angles, which primarily determine the prescribed coiling curvature. As noted earlier, the self-linking value is approximately conserved due to the interchange between $Wr$ and $Tw$, even in a highly tangled example. Therefore, this metric does not effectively reflect the degree of entanglement and is excluded from the performance score. We then design the score using mutual-link and object-link. As these metrics share the same topological interpretation and units, they are blended with equal weighting (50% : 50%).

The *object-linking* map (Fig. S3b) shows the direct interaction with specific target object, while the *mutual-linking* map (Fig. S3c) captures the overall complexity of inter-tentacle entanglement. Although high mutual linking does not always correspond to immediate contact with the current object, it indicates greater grasping possibility for more general object geometries. The combined score, therefore, balances object-specific grasp success with the potential for robust, morphology-driven grasping.

Another candidate metric is the interaction force measured on the fixed rigid rod, which functions analogously to a haptic force gauge. We compute the magnitude of the net contact force applied to the object. The resulting map (Fig. S3d) also reflects the level of interaction between the tentacles and the object. However, this force-based metric is not included in the final score for two primary reasons. First, the force magnitude is biased by radius and bending rigidity. Here, tentacles with less taper and higher stiffness at the distal part produce higher contact force. Second, the contact force is strongly correlated with object linking, which would overweight object-specific interactions if included.

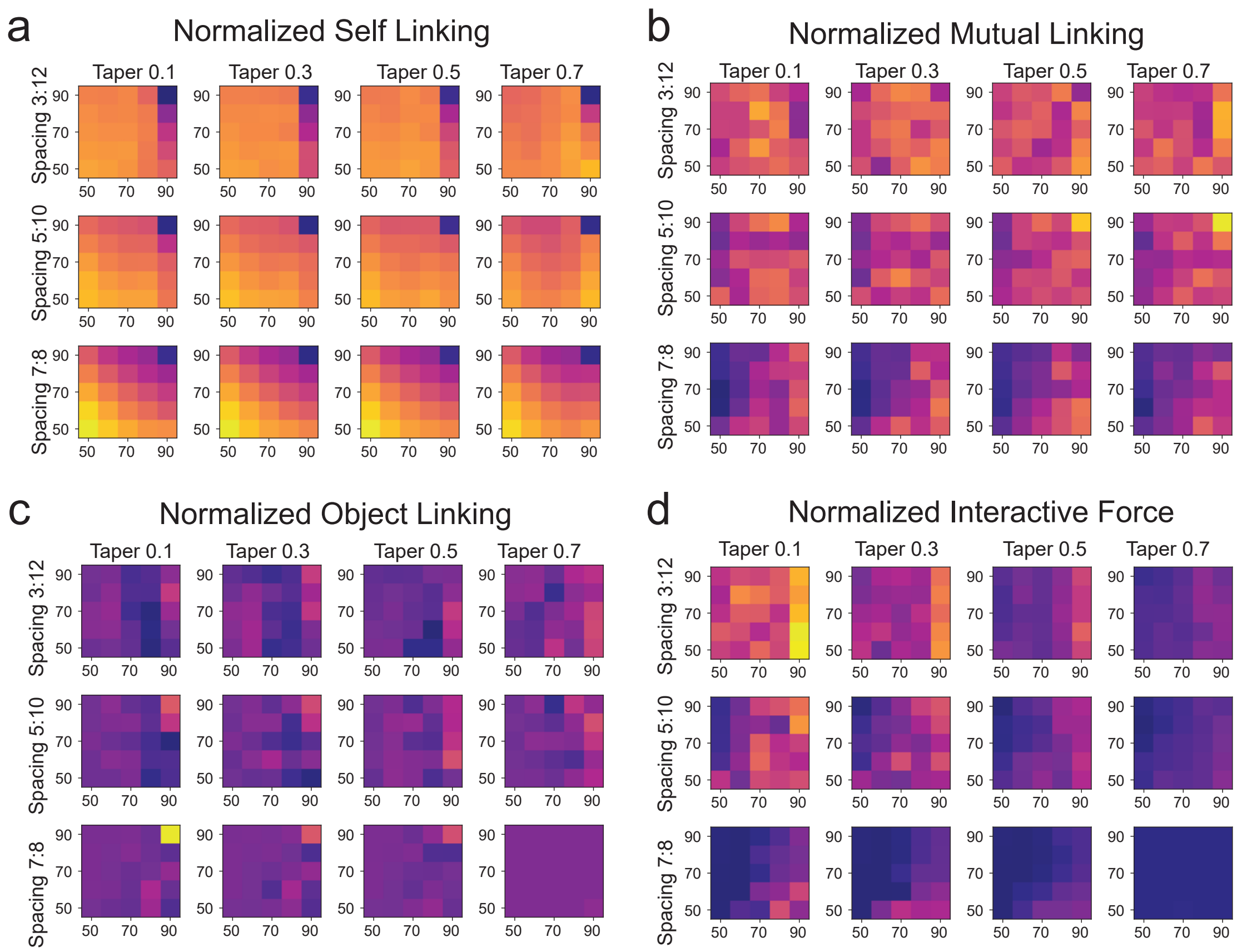


Figure S3: **Single evaluation metrics for zero gravity parameter sweep.** (a) Normalized average self-link numbers. (b) Normalized average mutual-link numbers. (c) Normalized average object-link numbers. (d) Normalized interaction force magnitude measured on the rigid target object

### Section 4. Tentacle behaviors in Air (w/gravity)

As in the observed tentacle behaviors on a flat surface in the main text, tentacle behaviors were also observed in air to assess for noticeable differences. The base unit cell tentacle design performed similarly to the gravity-assisted trial, seeing higher density shrinkage towards the proximal region of the tentacle. Adding a taper of 5° altered the folding density, seeing it transfer to the distal region of the tentacle. Introducing unequal spacing $b \neq c$ on a flat surface creates in-plane bending, but contrastingly in air creates a tight three-dimensional spiral due to the effect of gravity on the tentacle. Altering $\beta$ from 90° to 75° creates out-of-plane bending, and in-air, similarly creates a curvature and twisting biased coil.

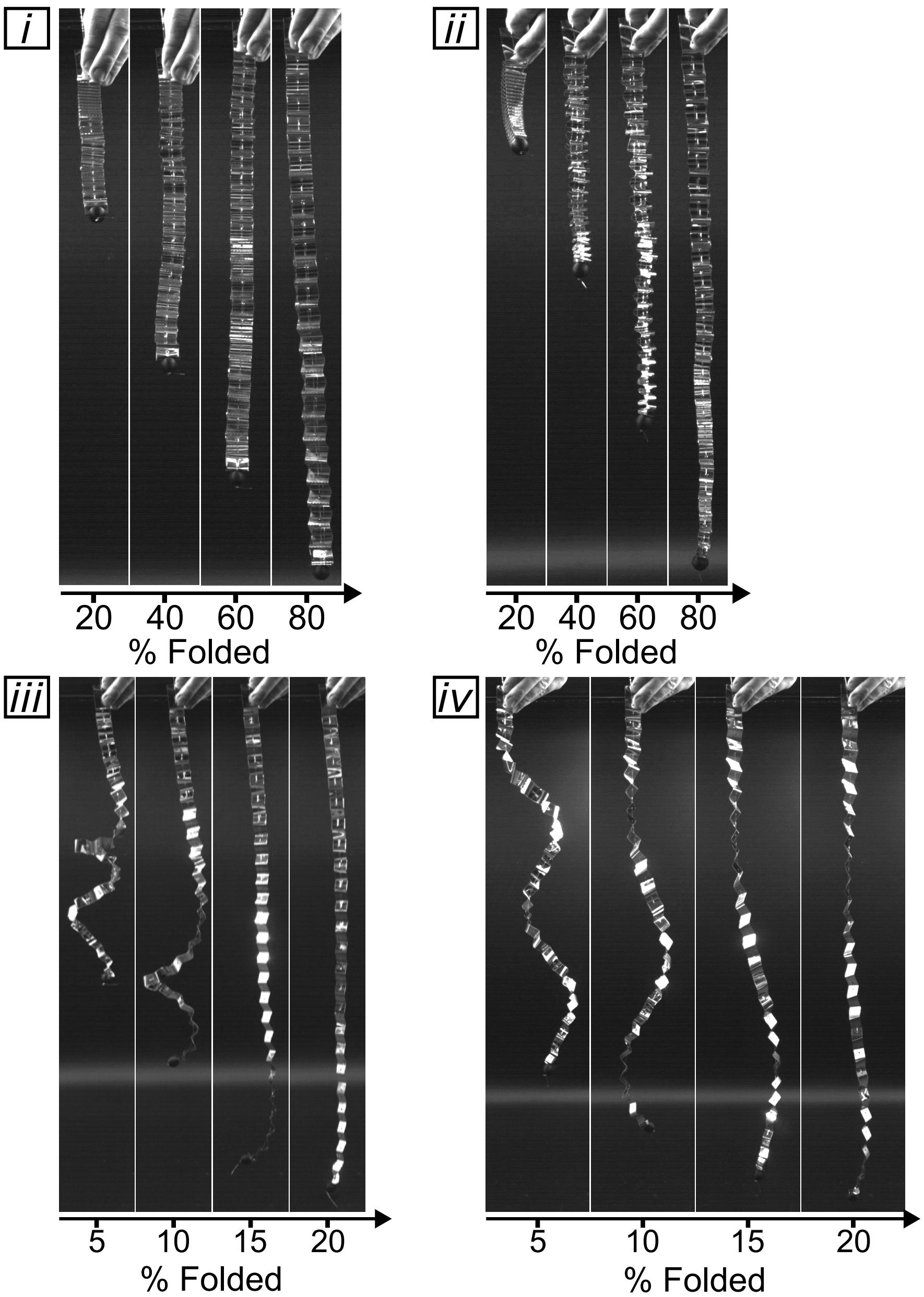


Figure S4: **Tentacle behavior testing in air under a high-speed camera.** (i) Base tentacle design with $\alpha = \beta = 90°$, $\theta = 0°$ and $b = c$. (ii) Tentacle design with $\alpha = \beta = 90°$, $\theta = 5°$ and $b = c$. (iii) Tentacle design with $\alpha = \beta = 90°$, $\theta = 5°$ and $b \neq c$. (iv) tentacle design with $\alpha = 90°$, $\beta = 75°$, $\theta = 5°$ and $b \neq c$.

### Section 5. Two-Tentacle Experimental Result

The two-tentacle test isolates the mechanical effect of entanglement by comparing actuation responses with and without inter-tentacle tangling. When the tentacles entangle, the force–actuation curve (red) departs clearly from the non-entangled baseline (orange), indicating strong mechanical coupling through contact constraints and frictional locking. In the non-entangled case, the tentacles fold largely independently, and the response follows a smoother, more repeatable trajectory. By contrast, entanglement introduces additional resistance and path dependence because deformation in one tentacle must occur against constraints imposed by the other, leading to a distinct global stiffness and transition behavior that cannot be inferred from single-tentacle mechanics alone.

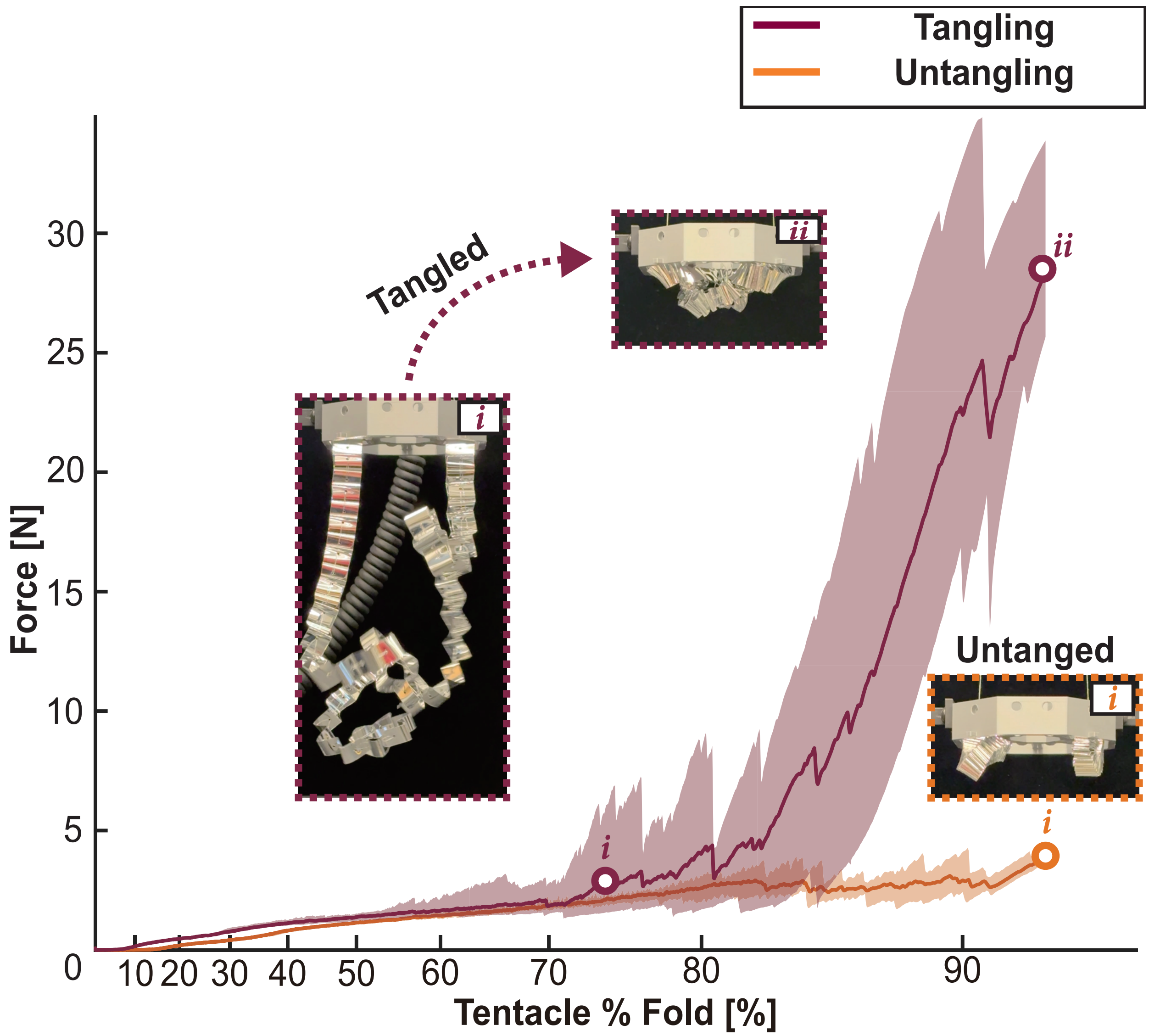


Figure S5: **Two-tentacle entanglement test.** Tendon force–actuation curves measured during simultaneous actuation of two tentacles in an entangled condition (red) and a non-entangled condition (orange).

**Section 6. Four Tentacle Experimental Result**

The four-tentacle experiment further shows that the system response depends not only on whether tangling occurs, but also on the specific tangling configuration that forms. The untangled case produces a response curve that is clearly separated from all tangled cases, reflecting near-independent folding with minimal mutual constraint. In contrast, each tangled curve corresponds to a different braiding or wrapping pattern among the tentacles, and the resulting spread of curves highlights the stochastic nature of entanglement formation. Importantly, these tangled states are not simply noisy variants of one behavior. Each tangling pattern exhibits its own mechanical signature, implying distinct effective stiffness and locking characteristics. Together, these results support the view that multi-tentacle grasping is governed by discrete, contact-driven entanglement states with different physical behaviors.

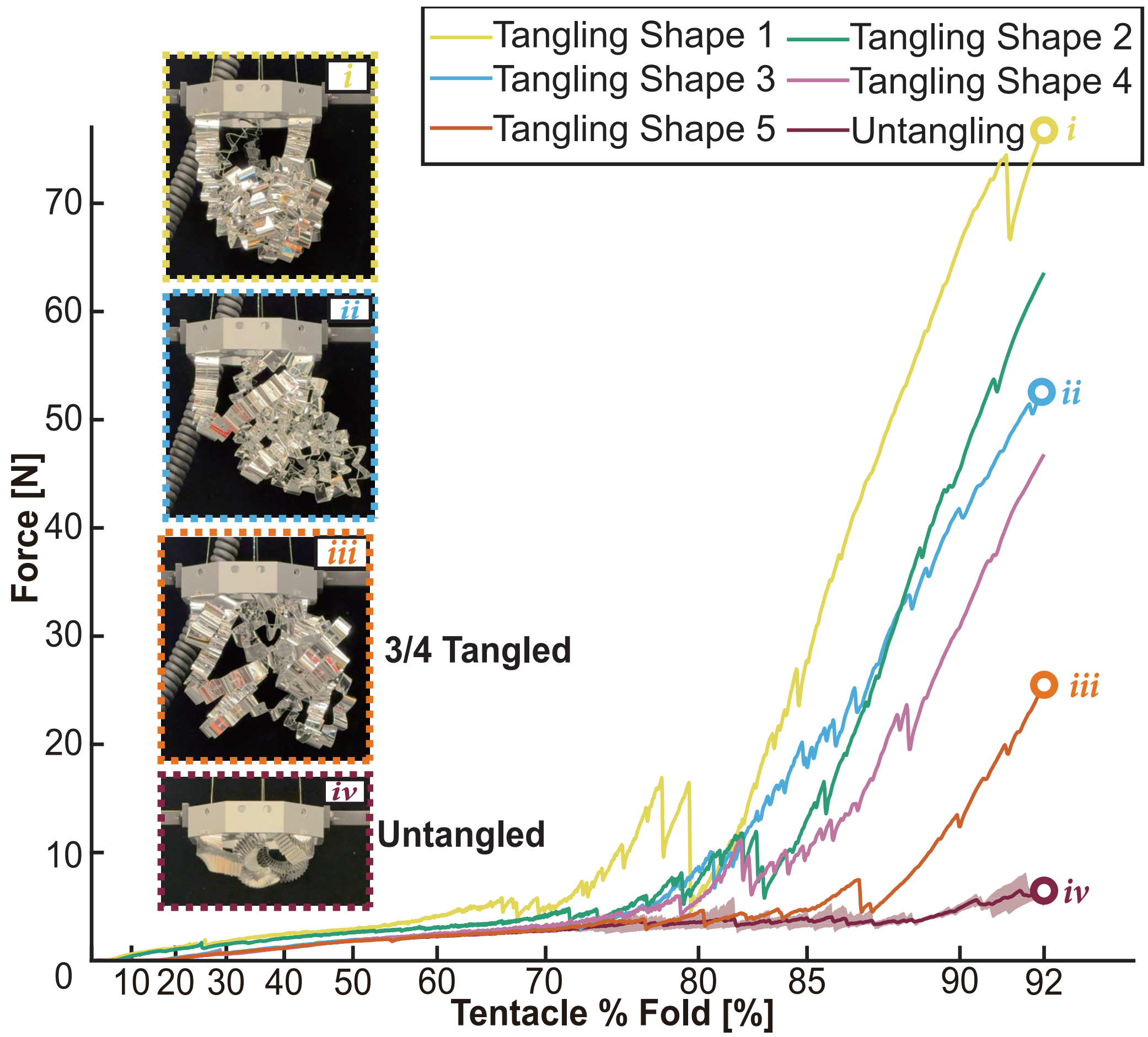


Figure S6: **Four-tentacle entanglement test.** Tendon force–actuation curves measured during simultaneous actuation of four tentacles in an untangled condition (baseline) and multiple tangled conditions.

## Section 7. Deployment Device Design

Figure S7 shows an exploded view of the stow-and-release deployment device used in the demonstration. The assembly is packaged to conform to a "1U" CubeSat form factor, so all tentacles are stored in a compact, launch-stowed configuration within the carrier volume. The assembly consists of an upper shell and lower shell (which together form the carrier and tentacle storage slots), and a central rotation shaft with eight radial branches. Each branch integrates a spring–push-pin module: the push pin is guided to slide radially, and a compression spring on the branch stores deployment energy while the pin is held inboard by the lower shell's inner surface. During deployment, rotating the shaft aligns each branch with a corresponding release slot in the lower shell; this alignment removes the geometric constraint, allowing the spring to expand and drive the push pin outward. The outward pin motion ejects the pre-coiled tentacle from its slot and initiates rapid unspooling, providing a compact, synchronized deployment mechanism without pneumatic plumbing or multi-channel actuation.

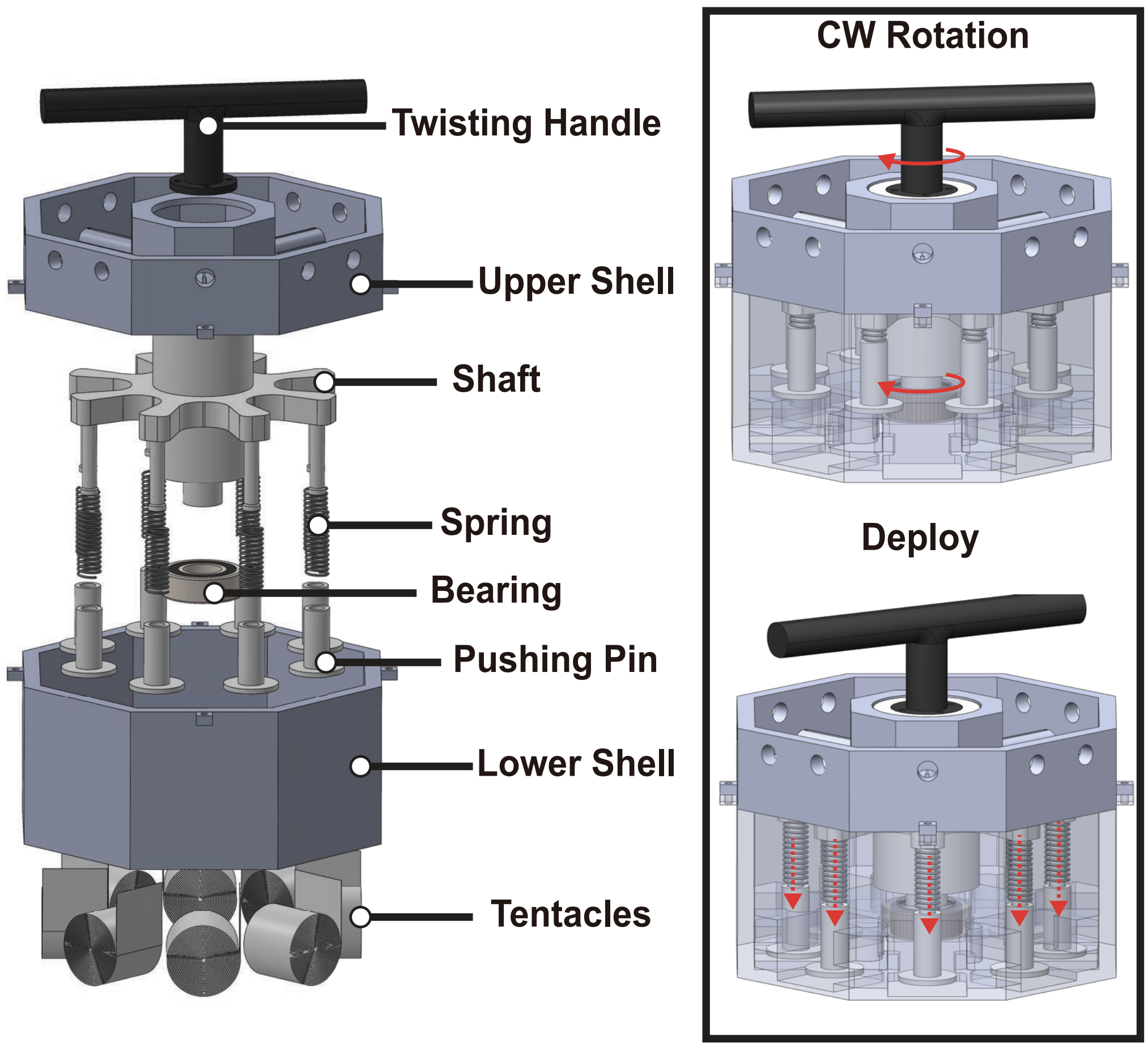


Figure S7: **Exploded view of the stow-and-release deployment device.**

## Section 8. Repeatability Test

The repeatability test (Movie S7) was performed to evaluate whether the tentacle gripper can maintain its grasping and release capability after repeated tendon-driven actuation. In this test, the tentacle was actuated continuously for 211 cycles. During cycle 1, the tentacle grasped and released the target object. Cycles 2 through 100 consisted of repeated actuation and reversal without an object, allowing the folding and recovery behavior of the tentacle to be examined under continuous operation. The tentacle

then successfully grasped and released the object again at cycle 101, followed by another 99 actuation-reversal cycles without an object. Finally, from cycle 201 to cycle 211, the tentacle successfully grasped the object in consecutive trials.

This result shows that the tendon-driven folding, coiling, and release process remained functional over repeated operation after optimization of the experimental setup. Importantly, the tentacle did not rely on a single prescribed contact point or deterministic wrapping path to recover grasping performance. Instead, the compliant origami architecture repeatedly generated object-linking configurations through folding-induced coiling and contact formation. The repeatability test, therefore, supports the robustness of the actuation and release mechanism over multiple cycles, while also providing experimental evidence that the gripper can recover its grasping function after repeated deformation.

## Section 9. Disturbance Test

The disturbance test (Movie S8) was conducted to examine the retention stability of the tentacle gripper under external perturbations after a grasp had formed. In this test, the tentacle first grasped the target object and maintained the grasp for 60 s. During this holding period, the object was struck 15 times using a metal bar from four different directions. The object remained retained throughout the test, indicating that the grasping state was able to resist repeated disturbances from multiple loading directions.

During the disturbance test, some local object links were observed to slip or fail when the object was tapped or struck. However, these local failures did not lead to global grasp failure. This behavior highlights an important feature of the proposed grasping mechanism: the grasp is not maintained by a single contact or a single locked configuration, but by multiple redundant object links generated through coiling, tangling, and frictional contact. As a result, when one or several local links are disrupted by impact, the remaining links can continue to constrain the object and preserve the overall grasp. This experiment, therefore, provides direct evidence that the robustness of the tentacle gripper arises from redundant, contact-driven object linking rather than from precise position control or rigid enclosure.

### Supplementary videos

**Movie S1:** High-speed tabletop actuation of a single tentacle, showing tendon-driven folding and deformation progression. 'Animated version of Figure 2d.'

**Movie S2:** High-speed in-air actuation of a single tentacle under gravity, highlighting transient coiling and bending during tendon retraction. 'Animated version of Figure S4.'

**Movie S3:** Emergent stochastic entanglement test by varying the number of active tentacles, illustrating pass/fail outcomes as redundancy decreases. 'Animated version of Figure 3a.'

**Movie S4:** Simulation visualization for multi-tentacle grasping under gravity. 'Animated version of screenshot 1-6 in Figure 3d.'

**Movie S5:** Tabletop grasping demonstration showing repeated release, repositioning, and regrasping of the same object during continuous operation. The gripper further demonstrates grasping of challenging objects, including a small smooth balloon, a complex irregular head massage tool, and a half-filled coffee cup without spilling. 'Animated version of Figure 4a.'

**Movie S6:** In-air balloon grasping comparison between design 1 ($\alpha$=$\beta$=90°) and design 2 ($\alpha$=90°, $\beta$=75°) using balloon sizes 3 and 4, highlighting differences in wrapping and retention. 'Animated version of Figure 4c & e.'

**Movie S7:** Repeatability test of the tentacle gripper over 211 actuation cycles, showing successful grasp-and-release at cycle 1 and cycle 101, repeated actuation and reversal without an object during cycles 2-100 and 102-200, and consecutive successful grasping during cycles 201-211.

**Movie S8:** Disturbance test of the tentacle gripper during object retention, showing the gripper holding the object for 60 s while the object is struck 15 times from four directions with a metal bar. The test highlights that even when some local object links fail during tapping or impact, the grasp remains stable through multiple redundant coiling and tangling links.

**Movie S9:** Underwater grasping demonstration showing successful target capture and retention under buoyancy-reduced gravity effects. 'Animated version of Figure 5.'

**Movie S10:** Simulation visualization for multi-tentacle grasping without gravity. 'Animated version of screenshot 1-4 in Figure 6b.'

**Movie S11:** Stow-and-release underwater deployment demonstration, showing the compact storage state, triggered release, and rapid tentacle deployment followed by capture. 'Animated version of Figure 6d.'
